# Supplementary material for: Self-supervised learning to predict intrahepatic cholangiocarcinoma transcriptomic classes on routine histology
Source: JHEP Rep. 2025 Nov 11;8(2):101675. doi: 10.1016/j.jhepr.2025.101675 (PMC12800354; doi:10.1016/j.jhepr.2025.101675)

Self-supervised learning to predict intrahepatic cholangiocarcinoma transcriptomic classes on routine histology

Aurélie Beaufrère, Tristan Lazard, Rémy Nicolle, Gwladys Lubuela, Jérémy Augustin, Miguel Albuquerque, Baptiste Pichon, Camille Pignolet, Victoria Priori, Nathalie Théou-Anton, Mickael Lesurtel, Mohamed Bouattour, Kévin Mondet, Jérôme Cros, Julien Calderaro, Thomas Walter, Valérie Paradis

Table of contents

Material and methods 2

Table S1 3

Table S2 4

Table S3 5

Table S4 6

Table S5. 7

Fig. S1 8

Fig. S2 9

Fig. S3. 10

Fig. S4 11

Material and methods

Slide preprocessing and tessellation

Slides from the discovery set were stained with HES and encoded in svs format. Slides from the external French validation set were stained with HES and encoded in ndpi format. Slides from the TCGA validation set were stained with hematoxylin-eosin (HE) and encoded in svs format. Tissue regions automatically extracted using Otsu thresholding were then exhaustively split into 2899811 patches of 224×224 pixels (without overlapping) at 10x using the OpenSlide library in Python.

We present the results in the discovery set according to the following three pre-processing protocols with or without extraction of the region of interest (ROI), each requiring varying levels of expert pathologist involvement (Figure 1, Fig. S3):

1. No-filter (∅): all tiles including tumour and non-tumour are processed as they are (encompassing both tumour and non-tumour regions).

2. Manual-filter (M): an expert pathologist (AB) extensively annotates tumour regions using ImageScope software, from which patches are extracted.

3. Learning-filter (A): tiles are filtered using logistic regression trained on a dataset of 3000 tile embeddings, randomly extracted and labelled by an expert pathologist (AB).

Table S1. List of morphological criteria assessed by the expert pathologist for all cases of the three datasets

Histological criteria	Assessment
**Tumour grade**	Classification into well, moderately or poorly differentiated tumour according to the 5th edition of the WHO classification
**Tumour histological type**	Small duct, large duct or other subtypes
**Necrosis**	Percentage
**Tumour fibrosis**	Semi quantitatively assessed, classified into three classes: no or mild, moderate and intense
**Immune tumour infiltration**	Semi quantitatively assessed, classified into four classes: no inflammation, low, moderate and high
**Tertiary lymphoid structure (TLS)**	Presence or absence

Table S2. Repartition of the five transcriptomic classes in the discovery set and in the two external validation sets.

Transcriptomic groups	Total n=307 (%)	Discovery set n=246 (%)	French external validation set n = 32 (%)	TCGA set n = 29 (%)
**Hepatic stem-like**	121 (39)	90 (37)	14 (44)	17 (59)
**Desert like**	14 (5)	11 (4)	1 (3)	2 (7)
**Tumor classical**	37 (12)	34 (14)	3 (9)	0 (0)
**Immune classical**	69 (22)	57 (23)	7 (22)	5 (17)
**Inflammatory stroma**	66 (21)	54 (22)	7 (22)	5 (17)

Table S3. Repartition of the five transcriptomic classes according to the type of samples in the discovery set.

Transcriptomic groups	Total n=246 (%)	Surgical samples n=109 (%)	Biopsies n = 137 (%)	*p*
**Hepatic stem-like**	90 (37)	53 (49)	37 (27)	**<0.001**
**Desert like**	11 (4)	4 (4)	7 (5)	0.759
**Tumor classical**	34 (14)	16 (15)	18 (13)	0.853
**Immune classical**	57 (23)	15 (14)	42 (31)	**0.002**
**Inflammatory stroma**	54 (22)	21 (19)	33 (24)	0.439

Table S4. Model performance for the hepatic stem-like binary classification task on external validation sets

Validation dataset	AUC score	Balanced accuracy score	F1 score
**TCGA set**	0.76	0.73	0.74
**French external set**	0.86	0.73	0.71

Area under the curve, AUC; The cancer genome atlas, TCGA

Table S5. Effects of the ROI extraction method on the external validation performances for the Hepatic stem-like binary classification task.

The same method is applied on both the training and validation datasets. On the TCGA set, no distinct advantage is observed for method A over M or vice versa, as it varies based on the metric under consideration. In the French external set, manually segmenting the tumour seems to be advantageous. Nevertheless, in both datasets, using an ROI extraction method is more effective than not using any.

**Validation dataset**	**ROI extraction method**	**AUC score**	**Balanced accuracy score**	**F1 score**
**TCGA set**	ø	0.74	0.60	0.61
	M	0.76	**0.73**	**0.74**
	**A**	**0.82**	0.64	0.63
**French external set**	ø	0.78	0.71	0.68
	**M**	**0.86**	**0.73**	**0.71**
	A	0.8	0.73	0.72

Area under the curve, AUC; Learning-Filter, L; Manual-Filter, M; No-Filter, ∅; Region of interest, ROI

Fig. S1. Histological features of iCCA

A. Example of small duct type iCCA

B. Example of large duct type iCCA

C. Example of an iCCA with an abundant fibrous stroma

D. Example of an iCCA with an abundant inflammatory stroma containing tertiary lymphoid structures

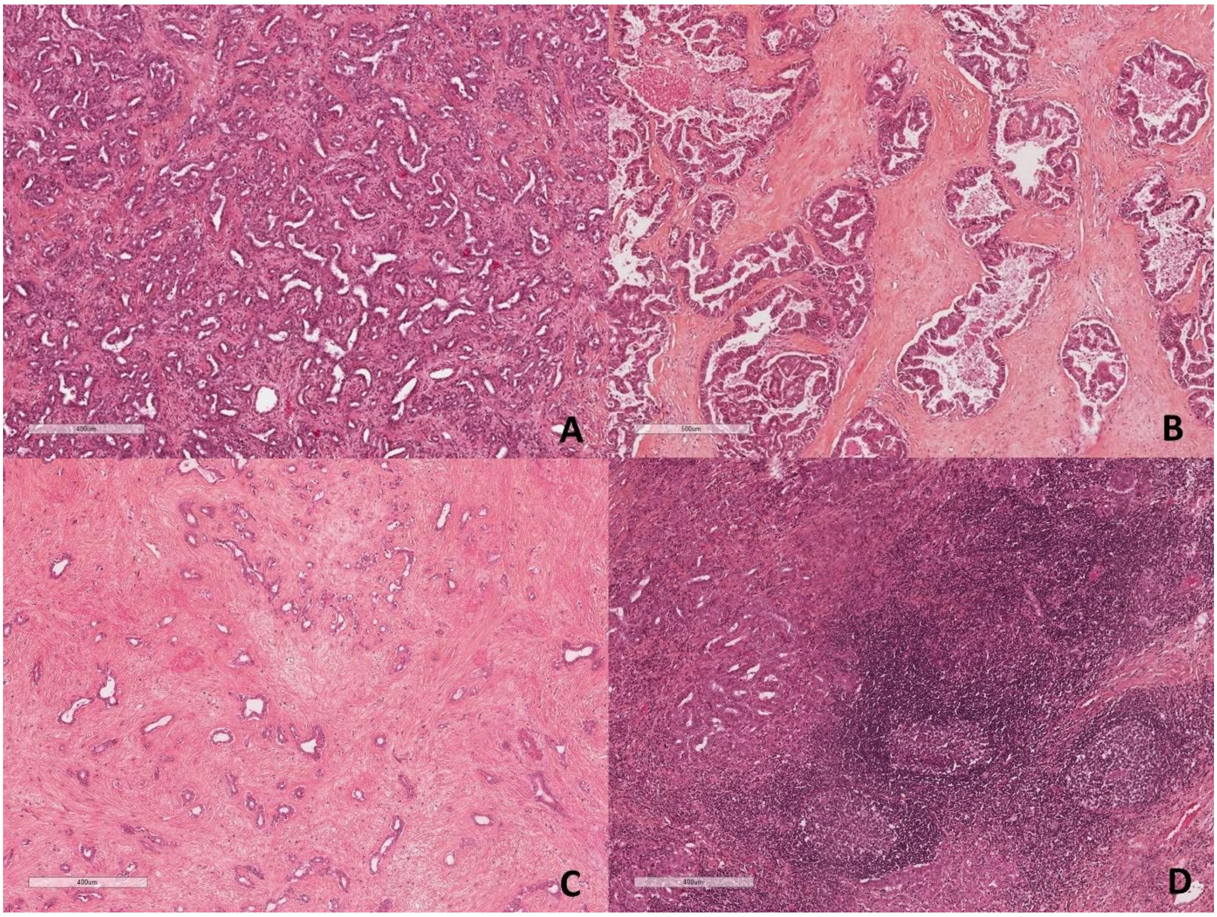

Fig. S2. Type of slides including in the study.

For surgical specimens, slides corresponding to blocks used for transcriptomic analysis in both the discovery set and the French external set were labeled as surgical slides (S RNA+). In the discovery and the TCGA sets, slides from blocks not used for transcriptomic analysis were labeled (S RNA-). For biopsy cases, the FFPE block used for RNA sequencing corresponded directly to the selected slide, which was labeled (B RNA+).

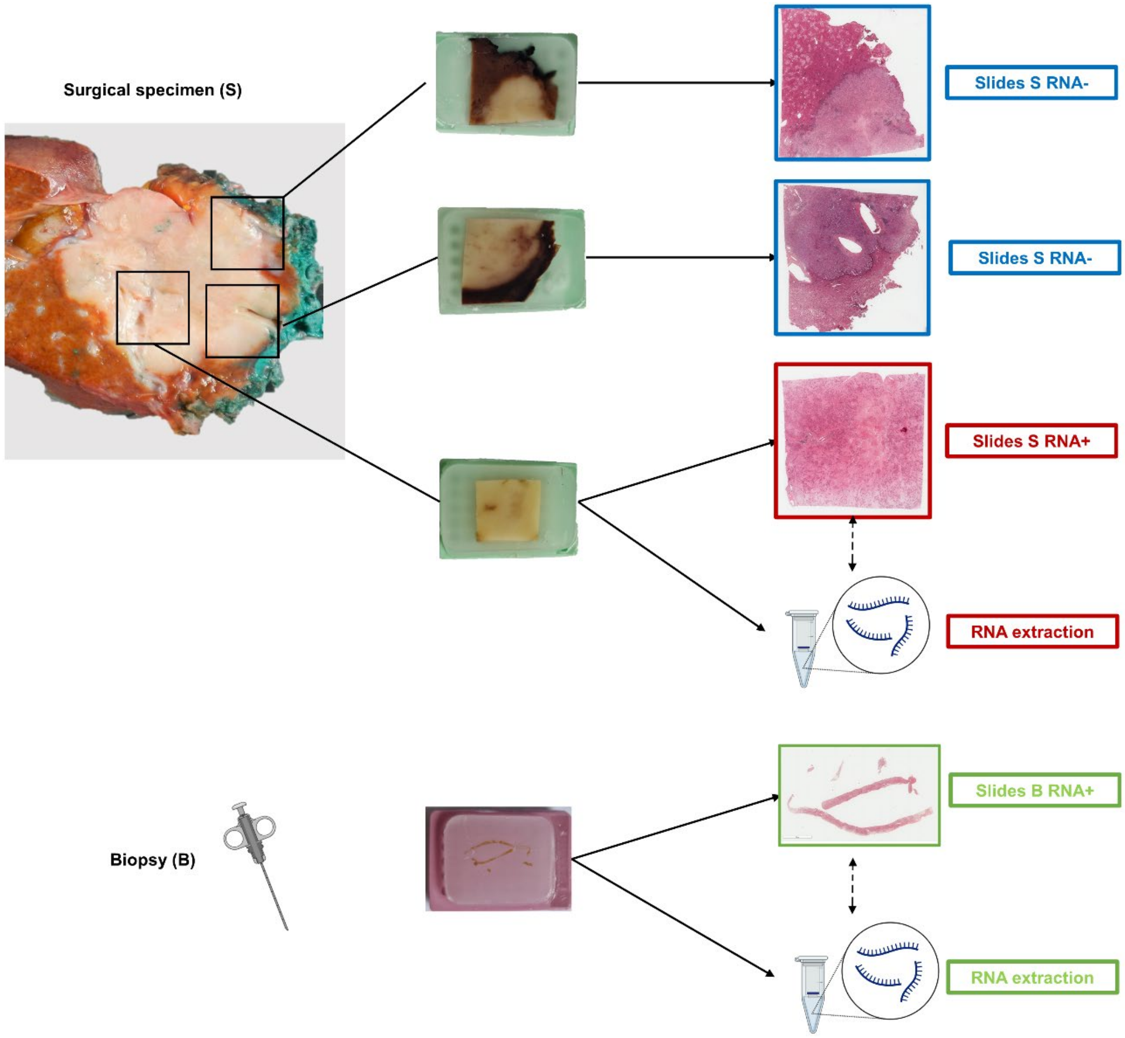

Fig. S3. Three different pre-processing protocols with or without extraction of region of interest (ROI).

- No-Filter (∅): All tiles including tumour and non-tumour are processed as they are, encompassing both tumour and non-tumour regions.
- Manual-Filter (M): An expert pathologist (AB) extensively annotates tumour regions using ImageScope software, from which patches are extracted.
- Learning-Filter (A): Tiles are filtered using a logistic regression trained on a dataset of 3000 tile embeddings, randomly extracted and labelled by an expert pathologist (AB).

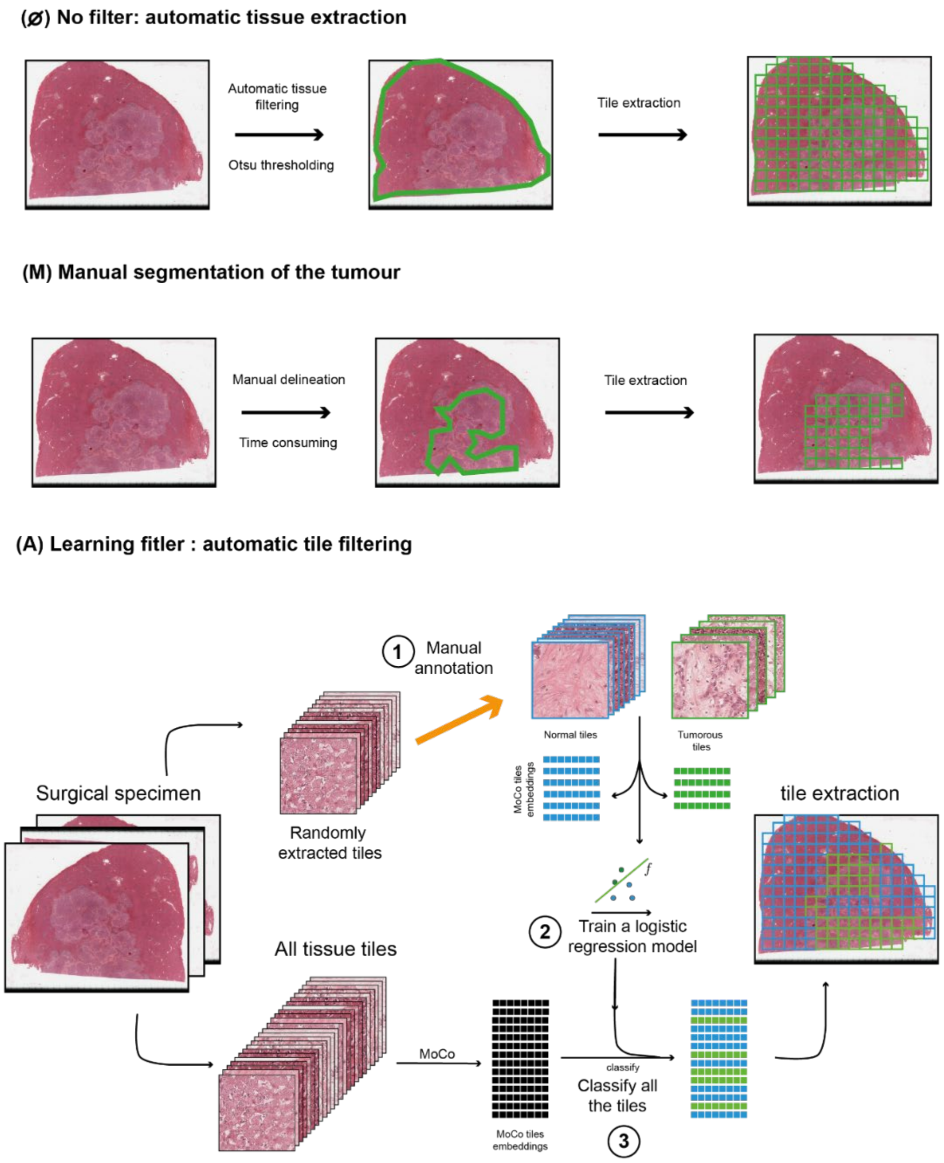

Fig. S4. Histological iCCA subtypes (large vs small duct) according to the five transcriptomic groups

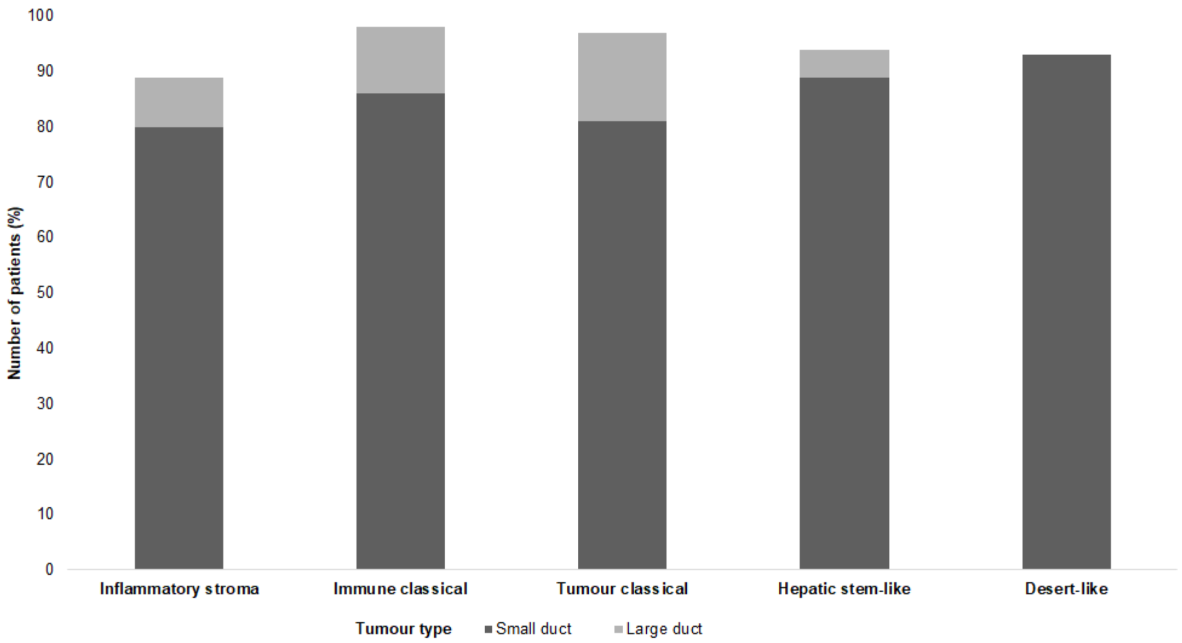

Supplement: Multimedia component 1 [file mmc1.pdf]
