# Supplementary material for: Self-supervised learning to predict intrahepatic cholangiocarcinoma transcriptomic classes on routine histology
Source: JHEP Rep. 2025 Nov 11;8(2):101675. doi: 10.1016/j.jhepr.2025.101675 (PMC12800354; doi:10.1016/j.jhepr.2025.101675)

Research article

JHEP|Reports

Self-supervised learning to predict intrahepatic cholangiocarcinoma transcriptomic classes on routine histology

Authors

Aurélie Beaufrère, Tristan Lazard, Rémy Nicolle, …, Julien Calderaro, Thomas Walter, Valérie Paradis

Correspondence

aurelie.beaufrere@aphp.fr (A. Beaufrère).

Graphical abstract

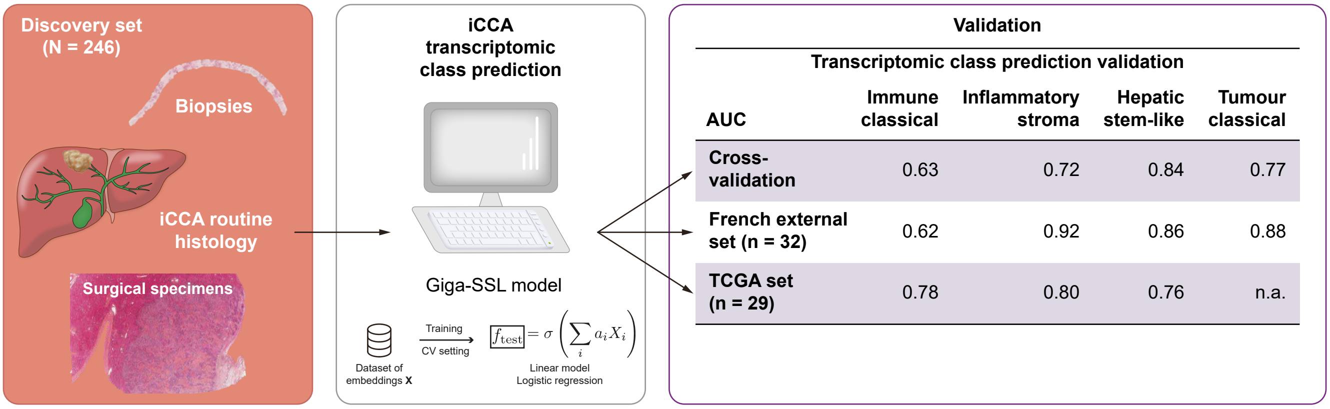

Highlights:

- Five transcriptomic classes of iCCA have been described, each associated with prognosis.
- We developed and validated an SSL model for predicting these transcriptomic classes.
- We obtained good performances for the prediction of the four most frequent classes.
- Our model could be used on routine slides of biopsy or surgical specimens.
- Our model could be used without the need for manual slide annotations.

Impact and implications:

Predicting transcriptomic classes directly from routine histological slides has the potential to enhance the clinical management of intrahepatic cholangiocarcinoma, enabling more accurate prognostication and supporting therapeutic decision-making. By eliminating the need for manual slide annotation, large tissue samples, or resource-intensive molecular analyses, our self-supervised learning-based model offers a practical and scalable solution that can be applied to both biopsy and surgical specimens. This approach could accelerate the adoption of transcriptomic classification in everyday practice and help guide more personalized treatment strategies for patients with intrahepatic cholangiocarcinoma.

https://doi.org/10.1016/j.jhepr.2025.101675
© 2025 The Author(s). Published by Elsevier B.V. on behalf of European Association for the Study of the Liver (EASL). This is an open access article under the CC BY license (http://creativecommons.org/licenses/by/4.0/). JHEP Reports, 2026, 8, 1–10

Self-supervised learning to predict intrahepatic cholangiocarcinoma transcriptomic classes on routine histology

Aurélie Beaufrère[1,2,3,*,†], **Tristan Lazard**[3,†], Rémy Nicolle[2], Gwladys Lubuela[2], Jérémy Augustin[4], Miguel Albuquerque[1,2], Baptiste Pichon[2], Camille Pignolet[2], Victoria Priori[2], Nathalie Théou-Anton[5], Mickael Lesurtel[6], Mohamed Bouattour[2,7], Kévin Mondet[1], Jérôme Cros[1,2], Julien Calderaro[4], Thomas Walter[3,‡], Valérie Paradis[1,2,‡]

JHEP Reports **2026**. vol. 8 | 1–10

Check for updates

Background & Aims: The transcriptomic classification of intrahepatic cholangiocarcinoma (iCCA) has recently been refined from two to five classes, each associated with pathological features, targetable genetic alterations, and survival outcomes. Despite its potential prognostic and therapeutic value, the transcriptomic classification is not routinely used in practice because of technical limitations, including insufficient tissue material and the high cost of molecular analyses. Here, we assessed a self-supervised learning (SSL) model for predicting iCCA transcriptomic classes on digitised whole-slide images (WSIs)

Methods: Transcriptomic classes defined from RNA sequencing data were available for all samples. The SSL method (Giga-SSL) was used to train our model on a discovery set of 766 WSIs from 137 biopsies and 109 surgical specimens obtained from 246 patients, using a five-fold cross-validation scheme. The model was validated in The Cancer Genome Atlas (TCGA) cohort (n = 29) and a French external validation set (n = 32), both using WSIs from surgical samples.

Results: The most frequent transcriptomic class was the hepatic stem-like class (37% [90/246] in the discovery set). Our model showed good to very good performance in predicting the four most frequent transcriptomic classes in the discovery set (AUC 0.63-0.84), especially for the hepatic stem-like class (AUC 0.84). The model performed equally well in predicting these transcriptomic classes in the two validation sets, with AUCs ranging from 0.76 to 0.80 in the TCGA set and 0.62 to 0.92 in the French external set.

Conclusions: We developed and validated an SSL-based model capable of predicting iCCA transcriptomic classes from routine histological slides of both biopsy and surgical samples. This approach may facilitate the clinical implementation of transcriptomic classification, improve prognostic assessment, and guide therapeutic decision-making in iCCA.

© 2025 The Author(s). Published by Elsevier B.V. on behalf of European Association for the Study of the Liver (EASL). This is an open access article under the CC BY license (http://creativecommons.org/licenses/by/4.0/).

Introduction

Intrahepatic cholangiocarcinoma (iCCA), the second most common primary liver cancers, is characterised by an increasing incidence worldwide and a poor prognosis.[1,2] Although surgery is the only curative treatment for iCCA, only 20-40% of patients can benefit from surgery because of diagnosis at an advanced stage.[3,4] For disease not amenable to surgery, systemic treatment – typically gemcitabine and cisplatin ± durvalumab or pembrolizumab in the first-line setting – is recommended, with a median overall survival of 12 months.[5–8]

Recent advances in the pathobiological and molecular understanding of iCCA have provided prognostic and theranostic factors. Transcriptomic analysis has initially identified two distinct groups of iCCA including an inflammatory class (40% of cases) characterised by activation of inflammatory signalling pathways, and a proliferation class (60% of cases) characterised by the activation of oncogenic signalling pathways.[9] Recently, this classification has been refined into five classes. The inflammatory class has been subdivided into two subclasses (inflammatory stroma and immune classical) and the proliferative class into three subclasses (hepatic stem-like, tumour classical and desert-like). Importantly, this classification has been associated with tumour microenvironment characteristics, genetic alterations and prognosis, that may guide the treatment strategy.[10] In particular, the most frequent transcriptomic class, the hepatic stem-like class, has been associated with a better prognosis and targetable genetic alterations including *IDH1* (isocitrate dehydrogenase 1) mutations (observed in 16% of hepatic stem-like cases compared

* Corresponding author. Address: Department of Pathology, Beaujon Hospital, 100 boulevard du Général Leclerc, Clichy, 92110, France; Tel.: +33 1 40 87 54 63; Fax: +33 1 40 87 00 77.
E-mail address: aurelie.beaufrere@aphp.fr (A. Beaufrère).
† These authors contributed equally to this work and share first authorship
‡ These authors contributed equally to this work and share last authorship
https://doi.org/10.1016/j.jhepr.2025.101675

to 7% in other classes), and *FGFR2* (fibroblast growth factor receptor 2) fusions (observed in 13% of hepatic stem-like cases compared to 5% in other classes). To note, specific inhibitors (ivosidenib and pemigatinib, respectively) have been approved by the US Food and Drug Administration as second-line treatments for locally advanced or metastatic iCCA.[11–13] Moreover, the two inflammatory subclasses may particularly benefit from immunotherapy. Despite its potential relevance, this comprehensive transcriptomic classification is not used in routine practice, mainly because informative data are based on expensive molecular techniques requiring adequate tissue samples rich in tumour cells.

Artificial intelligence (AI) models, particularly deep neural networks, are rapidly emerging in imaging.[14–17] With the development of digital pathology and wide access to digitised whole slide images (WSIs), AI approaches can be used for various classification tasks, such as diagnostic tasks.[18] For instance, AI approaches have demonstrated strong performance in identifying prognostic microscopic features and predicting transcriptomic classifications in hepatocellular carcinoma.[19–23] However, such deep learning techniques require large datasets (>1,000 slides)[24] and heavy computational machinery that limits in-depth studies on stratified datasets. We recently introduced Giga-SSL, a self-supervised learning (SSL) algorithm designed to generate generalist low-dimensional feature vectors of WSIs, which offers both computational efficiency and label-efficiency.[25]

The main objective of the present study was to predict iCCA transcriptomic classes on WSIs using the Giga-SSL model, with a focus on identifying the most frequent class, namely the hepatic stem-like class.

Patients and methods

Patient samples

The workflow of the study is summarised in Fig. 1. For the discovery set, we selected 246 formalin-fixed paraffin-embedded (FFPE) iCCA cases (109 surgical specimens and

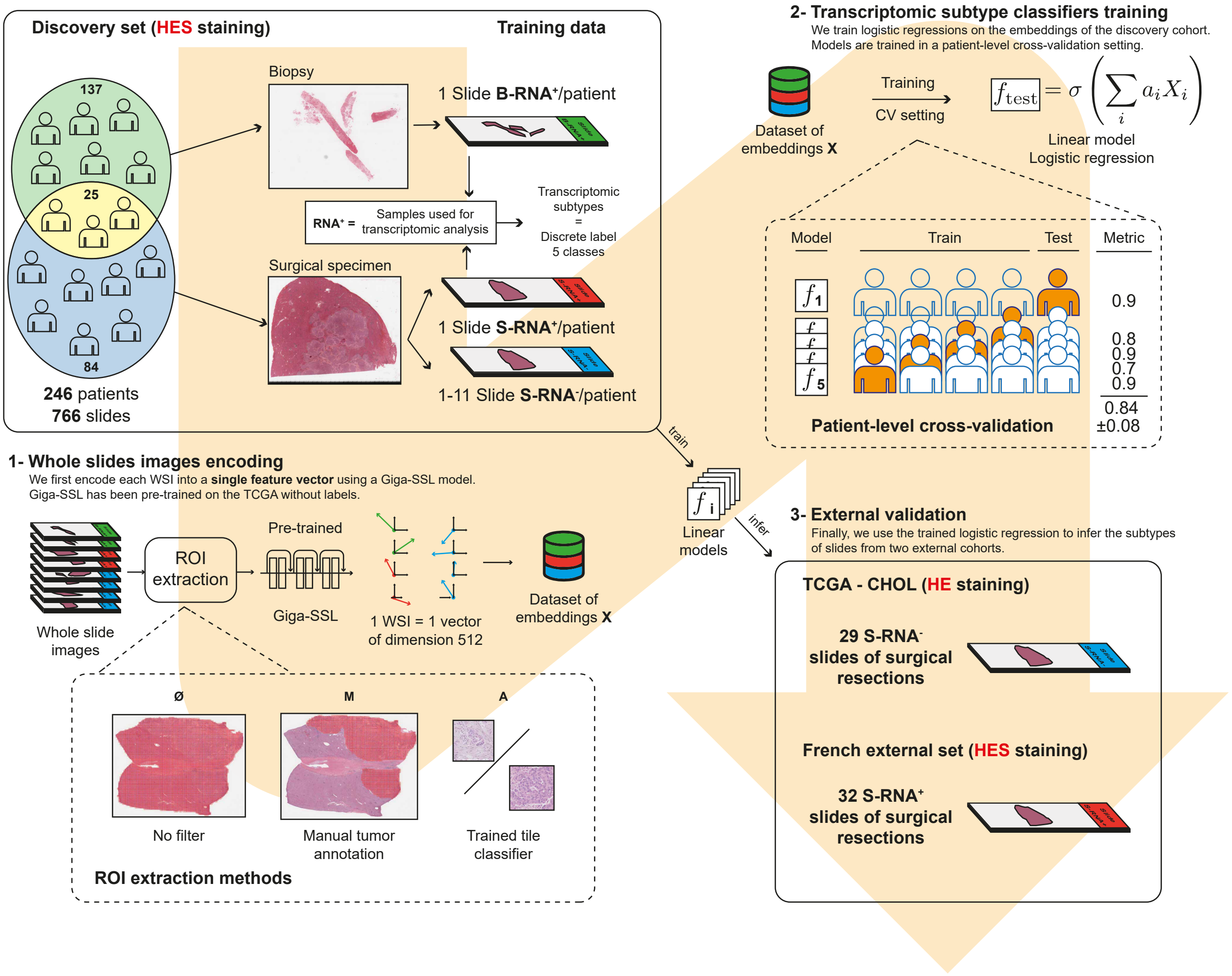

Fig. 1. Flowchart of the study. CV, cross validation; FFPE, formalin-fixed paraffin-embedded; HES, hematein eosin saffron, HES; ROI, region of interest; slide B RNA+, slide from biopsy sample corresponding to the sample used for the transcriptomic analysis; slide S RNA+, slide from surgical sample corresponding to the sample used for the transcriptomic analysis; slide S RNA-, slide from surgical sample, not corresponding to the sample used for the transcriptomic analysis; SSL, self-supervised learning; TCGA, The Cancer Genome Atlas.

137 biopsies) archived during 2000 and 2021 in the pathology department of Beaujon Hospital (Clichy, France). The material represented 766 hematein eosin saffron (HES) slides, divided into five folds at the patient level for cross-validation. All available slides for the surgical cases (including preoperative biopsies when available, n = 25) were selected for the study (median WSI per case: 5 [range 1-12]). The slides were scanned at 20x magnification with an Aperio scanner (ScanScope AT Turbo).

For the external validation sets, we used 29 iCCA cases (surgical FFPE samples; 29 WSIs) from The Cancer Genome Atlas cholangiocarcinoma (TCGA-CHOL) public dataset, and 32 surgical FFPE iCCA samples (32 WSIs, each representing the single most representative slide per case) from the Pathology Department of Henri Mondor Hospital (Créteil, France) as the French external validation set.

Written consent was obtained from all patients as required by French legislation. This study was approved by the local ethics committee (IRB 00006477 CER Paris Nord no. CER-2022-168).

The clinical and biological data recorded were age, sex, risk factors for iCCA, tumour size (radiological assessment for biopsy and pathological assessment for surgical specimens), number of tumours and overall survival.

Pathology review

All histological slides were reviewed by an expert liver pathologist (AB) and the assessed tumour features are listed (Table S1; Fig. S1). The stage of fibrosis in the non-tumoural liver when available was evaluated according to the METAVIR staging system.[26]

RNA sequencing

RNA extraction

RNA sequencing was performed for each case on one representative FFPE tumour block selected from the surgical specimen in both the discovery set and the French external set. Slides corresponding to these blocks (used for transcriptomic analysis) were labelled as surgical slides (S RNA+). In the discovery and the TCGA set, slides from blocks not used for transcriptomic analysis were labelled (S RNA-). For biopsy cases, the FFPE block used for RNA sequencing corresponded directly to the selected slide, which was labelled (B RNA+) (Fig. 1 and S2).

Briefly, 5 μm-thick sections, with macrodissection if necessary to retain only tumour areas, were cut from FFPE blocks. Total RNAs were further isolated by using the Qiagen FFPE RNA extraction kit (RNeasy FFPE kit, Qiagen) for the discovery set and the Recover AllTM Total Nucleic Acid Isolation Kit for the French external validation cohort (Invitrogen, Thermo Fisher Scientific).

Gene expression analysis

Gene expression was analysed using the SMARTer Stranded Total RNA-Seq Kit for the discovery set and QuantSeq 3' mRNA-seq Kit for the French external validation set. Only genes quantified in at least 50% of samples were retained for the analysis. RNA alignment was performed using a dedicated pipeline (https://github.com/GeNeHetX/RNApipeline). Reads are aligned with the reference genome ensemblv107_GRCh38.75 by STAR[27] and gene expression counts obtained with FeatureCount.[28] Gene expression profiles were quantile-normalized. The mean expression of each gene-set-defined gene signature was computed following a gene-wise centring in each dataset (without variance scaling). The transcriptomic class with the highest gene set-averaged expression was assigned to each sample. The same process was used with the TCGA dataset.

Slide pre-processing and tessellation

Slides from the discovery set were stained with HES and encoded in svs format. Slides from the external French validation set were stained with HES and encoded in ndpi format. Slides from the TCGA validation set were stained with H&E and encoded in svs format. Tissue regions automatically extracted using Otsu thresholding were then exhaustively split into 2,899,811 patches of 224 × 224 pixels (without overlapping) at 10x using the OpenSlide library in Python.

We present the results in the discovery set according to three pre-processing protocols with or without extraction of the region of interest (ROI), each requiring varying levels of expert pathologist involvement (Fig. 1 and S3):

1. No filter: all tiles including tumour and non-tumour are processed as they are (encompassing both tumour and non-tumour regions).
2. Manual filter: an expert pathologist (AB) extensively annotates tumour regions using ImageScope software, from which patches are extracted.
3. Learning filter: tiles are filtered using logistic regression trained on a dataset of 3,000 tile embeddings, randomly extracted and labelled by an expert pathologist (AB).

Machine learning algorithms

Data split

Training involved using a 5-fold cross-validation framework in the discovery set. Splits were stratified according to the output variable, at the patient level. They were shared among all trainings to ensure the fairness of comparison.

Giga-SSL representations

The Giga-SSL model was trained on a single V100 GPU on the TCGA-FFPE dataset following the training framework provided in our previous study[25] with the exception of the following:

- Training was performed for 100 h, or 7,800 epochs
- WSI embeddings were ensembled over 100 views, then L2-normalized.

Finally, we used L2-regularised logistic regressions (C=7, max_iter = 10,000, and class_weight set as "balanced") as end classification models.

MIL baseline algorithms

Beside the Giga-SSL-based classifications, we provided some baseline classification algorithms for comparison. They were based on the deep attention multiple instance learning (MIL)

algorithm introduced in the work of Ilse *et al.*[29] and slightly modified by Lazard *et al.*.[30] MIL models were trained from scratch and operate on tile embeddings extracted from the last layer of pretrained ResNet18.[31] We used ResNet18 pre-trained on imagenet and on the TCGA with MoCo (*i.e.* the tile-encoder used in the Giga-SSL model).

External dataset inference

The probabilities predicted by the five logistic regression models trained on the training set (each corresponding to a training fold) were averaged, and performance metrics were computed using these pooled probabilities.

Statistical analysis

Continuous variables were compared using Student's *t* test, and categorical variables were compared using the chi-squared or Fisher's exact tests. Survival curves were estimated by the Kaplan-Meier method and compared with log-rank statistics. $p < 0.05$ was considered statistically significant (SPSS software). The performance of AI models was assessed using the AUC score, balanced accuracy score and F1 score (macro-average).

Results

Patient characteristics

The main clinical and pathological features of patients and tumours for each dataset are presented in Table 1. At the pathological level, the French external validation set had higher proportions of large duct type and marked immune tumour infiltration than the two other sets (22% *vs.* 7% and 3%, $p = 0.018$; 59% *vs.* 38% and 33%, $p = 0.011$).

Transcriptomic classes

The proportion of each transcriptomic class in the three datasets is represented in Fig. 2 and Table S2. The most frequent transcriptomic class was the hepatic stem-like class (37% [90/246] in the discovery set, 44% [14/32] in the French external validation set, and 59% [17/29] in the TCGA validation set). The desert-like class was very rare (4% [11/246] in the discovery set, 3% [1/32] in the French external set, and 7% [2/29] in the TCGA validation set). The distribution closely mirrored that reported in the Mount Sinai cohort by Martin-Serrano *et al.*[10] (Table S3). Of note, the distribution of the five transcriptomic classes differed between surgical and biopsy samples in the discovery set (Table S4). The hepatic stem-like class was more frequent in surgical than biopsy samples (49% *vs.* 27%, $p < 0.001$), whereas the immune classical class was more frequent in biopsy samples (31% *vs.* 14%, $p < 0.002$).

As expected, transcriptomic classes in all cohorts were associated with pathological features (Fig. 2B,C). Prominent tumour fibrosis was mainly observed in the inflammatory stroma class (38/66, 58%). High tumour immune infiltration was most prevalent in the inflammatory stroma and immune classical classes (19/66 [29%] and 8/69 [16%], respectively). Hepatic stem-like and desert-like classes exhibited low tumour immune infiltration (67/120 [56%] and 7/15 [47%]). The five

Table 1. Clinical and pathological features of the different datasets of the study.

	Discovery set, N = 246	French external validation set, n = 32	TCGA validation set, n = 29	*p* values
**Clinical features**				
Age, years (mean)	63 [27-88]	64 [25-85]	63 [29-82]	0.810
Sex (male/female)	141 (57)/105 (43)	23 (72)/9 (28)	13 (45)/16 (55)	0.099
**Risk factors**				
HBV	28 (11)	3(9)	NA	0.734
HCV	16 (7)	0 (0)	NA	0.231
MS	72 (30)	5 (16)	NA	0.141
Chronic alcohol intake	43 (18)	6 (19)	NA	0.859
PSC	4 (2)	3 (9)	NA	**0.035**
Other	11 (4)	1 (3)	NA	1.000
No risk factor	72 (29)	17 (53)	NA	**0.003**
**Pathological features**				
Cirrhosis (F4 METAVIR)	24 (10)	4 (12)	NA	0.544
Multinodularity	67 (27)	10 (31)	NA	0.633
Size (mean, cm)	7 [1-22]	7 [1-16]	NA	0.604
Small duct type	214 (87)	22 (69)	27 (93)	**0.010**
Large duct type	18 (7)	7 (22)	1 (3)	**0.018**
Well differentiated tumour	82 (33)	15 (47)	12 (41)	0.253
Moderately differentiated tumour	135 (55)	14 (44)	14 (48)	0.426
Poorly differentiated tumour	29 (12)	3 (9)	3 (10)	1.000
Fibrosis (no or mild/moderate or intense)	49 (20)/197 (80)	4 (12)/28 (88)	6 (21)/23 (79)	0.723
Immune infiltration (no or low/moderate or high)	166 (67)/80 (33)	13 (41)/19 (59)	18 (62)/11 (38)	**0.011**
TLS	8 (3)	5 (16)	8 (28)	**<0.001**
Necrosis (median, %)	18 [0-90]	0 [0-60]	0 [0-30]	**<0.001**

Data are n (%) unless otherwise indicated.
NA, data not available.
MS, metabolic syndrome; PSC, primary sclerosing cholangitis; TLS, tertiary lymphoid structure; TCGA, The Cancer Genome Atlas.
If not available in the TCGA set, the statistical analyses were performed only between the discovery and the French external validation sets.
Continuous variables were compared using the Student's *t* test, and categorical variables were compared using chi-squared or Fisher's exact tests.

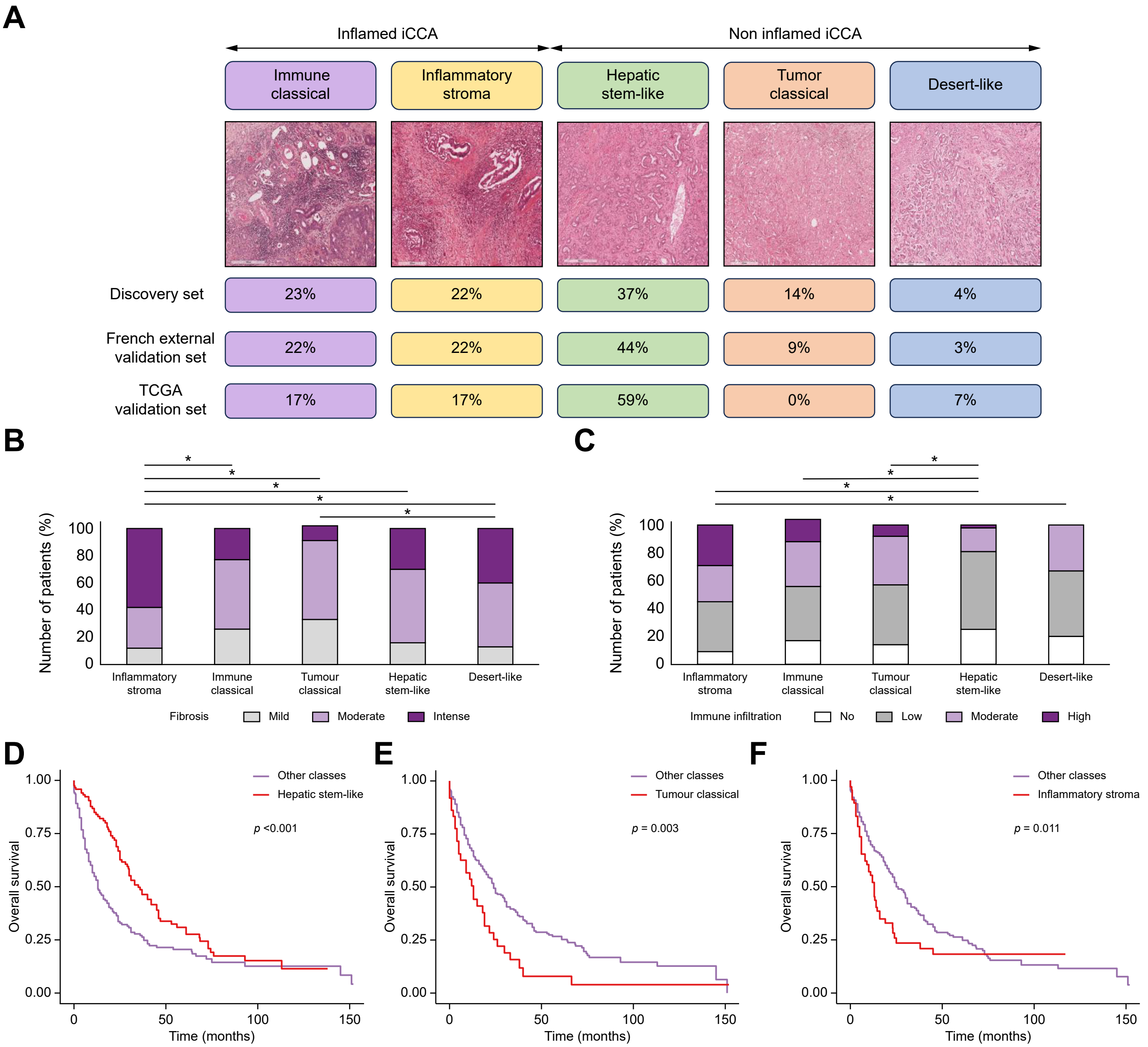

N° at risk				
Other classes	225	42	9	1
Hepatic stem-like	63	5	1	0

N° at risk				
Other classes	256	46	10	1
Tumour classical	32	2	1	1

N° at risk				
Other classes	225	42	9	1
Inflammatory stroma	63	5	1	0

Fig. 2. Distribution and characterisation according to histological features and overall survival of the five transcriptomic classes. (A) Distribution of each transcriptomic class according to the different datasets used in the study and representative histological images of each transcriptomic class (HES), (B) semi-quantitative assessment of the amount of tumour fibrosis in each transcriptomic class (*p <0.05), (C) Semi-quantitative assessment of the amount of tumour immune infiltration in each transcriptomic class (*p <0.05), Kaplan-Meier curves for overall survival with risk tables according to (D) hepatic stem-like, (E) tumour classical and (F) inflammatory stroma transcriptomic class. Continuous variables were compared using Student's t test, and categorical variables were compared using chi-squared or Fisher's exact tests. Survival curves were estimated by the Kaplan-Meier method and compared with log-rank statistics.

transcriptomic classes did not differ by tumour histological type (small *vs*. large duct, p = 0.074) (Fig. S4).

Clinically, overall survival (OS) was significantly associated with three transcriptomic classes in all cohorts. OS was improved in the hepatic stem-like class compared to the other transcriptomic classes (OS median: 49 *vs*. 35 months, hazard ratio [HR] 0.58; 95% CI 0.44-0.75; p <0.001) but was lower in the tumour classical and inflammatory stroma classes compared to the other classes (OS median: 21 *vs*. 43 months, HR 1.76; 95% CI 1.09-2.82; p = 0.003 and 31 *vs*. 43 months, HR 1.50; 95% CI 1.04-2.17; p = 0.011, respectively) (Fig. 2D-F).

Table 2. Cross-validated performance of the Giga-SSL and MIL models on the discovery cohort for the hepatic stem-like binary classification task according to three different pre-processing protocols.

Model	Tile filter	AUC score	Balanced accuracy score	F1 score
**Giga-SSL**	∅	0.8	0.72	0.72
	M	**0.84**	**0.76**	**0.76**
	A	0.82	0.74	0.75
**MoCo + MIL**	∅	0.74	0.67	0.67
	M	0.82	0.75	0.75
	A	**0.82**	**0.75**	**0.74**

A, learning filter; M, manual filter; ∅, no filter; MIL, multiple instance learning; SSL, self-supervised learning. The best results for each method are presented in bold.

Using self-supervised WSI representations for transcriptomic class prediction

We initially focused on the binary classification of the most frequent class (hepatic stem-like) before expanding our analysis to the other transcriptomic classes, excluding the desert-like class, which was too rare across the three datasets to develop a reliable predictive model.

Predicting the hepatic stem-like class

Discovery set

Table 2 shows cases of the cross-validated performances of both the Giga-SSL and MIL models in the discovery cohort for the hepatic stem-like binary classification task. The performance of the Giga-SSL model peaked when combined with manual tumour annotation, which resulted in a mean AUC of 0.84. Performance improved when a protocol of ROI extraction was applied to WSIs, regardless of whether the extraction of the tumour tiles was manual or learned. This improvement was particularly notable when using the classic MIL models, with the absence of WSI filtering leading to an 8-point decrease in AUC. For the Giga-SSL models, the absence of WSI filtering led to a 4-point decrease in AUC.

External validation

Table S5 presents the results of the external validation of models trained on all slides of the discovery cohort, with a manual filter applied to the WSIs. Logistic regression trained on the Giga-SSL embeddings of the discovery cohort demonstrated strong transferability to both the French external set (AUC = 0.86) and the TCGA set (AUC = 0.76). Notably, the TCGA cohort slides were stained with H&E, which differs from the staining protocol used for the discovery cohort's slides (HES) and further emphasizes the generalizability of the models.

Influence of the pre-processing ROI extraction protocol

We detailed in Table S5 the external validation results when models were trained according to the three different pre-processing protocols with or without ROI extraction. As observed in the cross-validated experiments on the discovery set, using an ROI extraction method (extraction only of the tumour tiles) was advantageous for both external datasets *vs.* extraction of both tumour and non-tumour tiles.

Effect of slide composition of the training set

In Fig. 1 and S2, the discovery dataset is illustrated to contain various types of slides, directly (RNA+) or indirectly associated with transcriptomic analysis (RNA-), including both surgical slides (S) and biopsy (B) slides. We aimed to determine how the composition of the training set affected the generalisation performance of classification models. For this, we trained models using training sets with different compositions of S RNA+, S RNA- and B RNA+ slides and monitored the

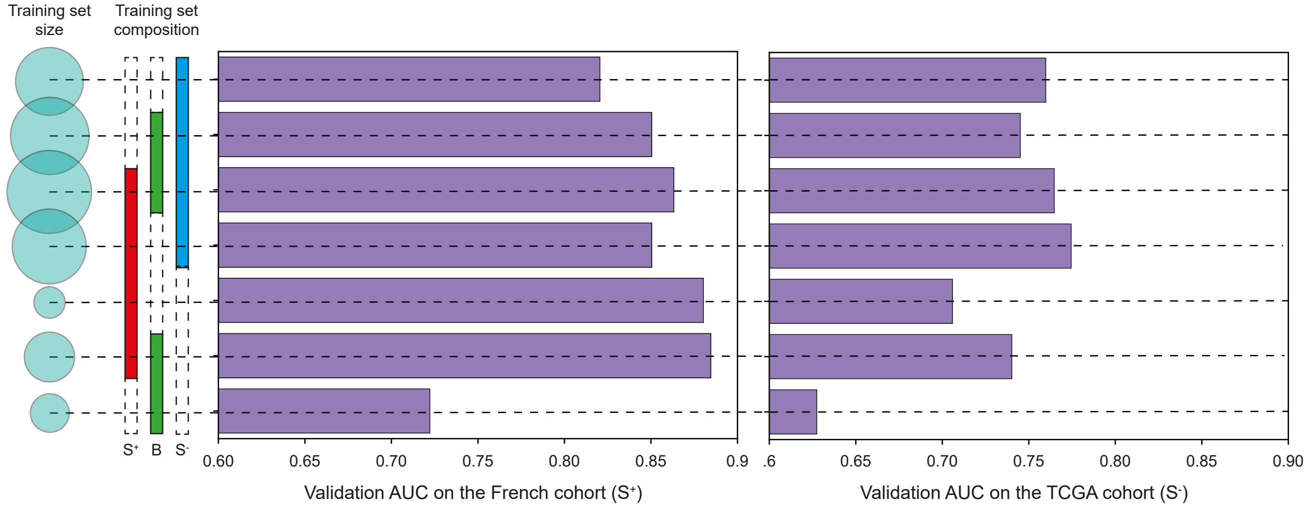

Fig. 3. Effect of the composition of the training set on the performance of the Giga-SSL model for the hepatic stem-like binary classification task. ROI, region of interest; slide B RNA+, slide from biopsy sample corresponding to the sample used for the transcriptomic analysis; slide S RNA+, slide from surgical sample corresponding to the sample used for the transcriptomic analysis; slide S RNA-, slide from surgical sample, not corresponding to the sample used for the transcriptomic analysis; SSL, self-supervised learning.

prediction performance on the two external validation sets. The results are provided in Fig. 3. For the French external validation set, the highest performance was achieved when the training set was limited to slides that were directly associated with the transcriptomic analysis (S RNA+ and B RNA+).

Next, we turned to the validation on the TCGA dataset, exclusively consisting of S RNA-slides (*i.e.* slides for which the transcriptome was analysed on a different tissue block). For this dataset with a putatively noisier ground truth, the model's performance seemed closely related to the size of the training data rather than its composition (Fig. 3).

Prediction of the other transcriptomic classes

We conducted analogous experiments for predicting other transcriptomic classes including inflammatory stroma, immune classical and tumour classical. As with the hepatic stem-like class, we trained binary classifiers for each class using a patient-level five-fold cross-validation setting and then applied them to the validation sets. We manually set the ROI method and trained the model using the complete dataset (S RNA+, B RNA+, S RNA-). Table 3 presents the results of these experiments. The three classes could be predicted in a cross-validation setting and demonstrated generalisation capabilities on the validation sets.

The classification task for the inflammatory stroma class outperforms the others on the validation set, achieving an AUC of 0.92 for the French set and 0.80 for the TCGA set. Despite the absence of tumour classical slides in the TCGA dataset, this class still seemed predictable and showed good generalisation with an AUC of 0.88 on the French set. However, despite some discernible signal for the immune classical class, it was the most challenging to classify. Confusion matrix of the misclassified cases in the whole validation set showed that among the slides wrongly classified as positive, most belong to the hepatic stem-like, immune classical, or inflammatory stroma classes (Table S7).

Discussion

We demonstrate in this study that prediction of iCCA transcriptomic classes, especially hepatic stem-like and inflammatory stroma classes, is achievable and effective using an SSL method applied to routine WSIs. This is particularly relevant as transcriptomic classes are associated with prognosis and expected to impact on the treatment response, particularly regarding immunotherapy and targeted therapies.[9,10] Furthermore, because the hepatic stem-like group is associated with targetable molecular alterations,[10] our model could be used as a pre-screening tool, since even though recent guidelines recommend their investigation, molecular profiling cannot always be carried out in routine practice, mainly for logistical and technical reasons.[6] We also confirmed in our cohorts the association between the two inflammatory classes and tumour microenvironment composition (inflammation and/or fibrosis) assessed by a pathologist on HES slides, which may benefit from immune checkpoint inhibitor treatments.[10] Consistent with these findings, we recently investigated the tumour microenvironment composition in a cohort of iCCA surgical specimens and showed that the inflammatory stroma class was associated with a CD8-rich infiltrate, whereas the immune classical class correlated with a low proportion of FAP+ fibroblasts.[32]

Currently, the leading methods for WSI classification rely on MIL.[29,33] However, annotated datasets are often small, typically a few hundred to a few thousand WSIs, which may result in overfitting and underperforming models, whereas large unannotated datasets of tens of thousands WSI are available. Here, we used a slide level SSL model, called Giga-SSL, allowing us to leverage the large number of WSIs without annotations to infer powerful slide representations.[25] Our model surpassed the performance of the standard MIL model in the binary classification task for the hepatic stem-like class. We observed a gain of 2 to 6 points in AUC according to the pre-processing ROI extraction method. This model significantly improves efficiency by increasing speed and reducing the use of computational resources. Creating WSI embeddings from raw SVS or TIFF images takes about 13 s (using the code from https://github.com/trislaz/Democratizing_WSI). After generating the WSI embeddings, all analyses were done with logistic regression and ran smoothly on a standard laptop CPU, showing very low computational demands — the full cross-validation and inference process takes only a few minutes, a major improvement compared to transcriptomic analyses, which can take several weeks.

Our model was trained and validated on three different sets to capture potential heterogeneity in tumour sampling (biopsy and surgical samples, whether or not directly associated with transcriptomic analysis) and staining methods (HE or HES). Indeed, despite being exclusively trained on HES slides, our models could predict the transcriptomic classes on either HES or H&E slides. This is particularly interesting because the routine staining differs among countries and laboratories, leading to colour heterogeneity that could affect the performance of the AI model.[34,35] In addition, and as expected, our model demonstrated better prediction when applied to tumour tiles rather than to all tiles (including non-tumour tiles), which suggests that the essential information regarding transcriptomic subclasses is contained in the tumour itself. To bypass the time-intensive process of manual annotations by a pathologist (which is a main limitation of using AI models), our data support the use of an automatic learning filter given its close performance to that when using a manual filter, which represents a favourable balance between time invested and classification performance.

Furthermore, we provide a comparative analysis that explores the influence of training set composition according to

Table 3. Prediction for the four most frequent transcriptomic classes according AUC scores.

Transcriptomic classes	CV results in the discovery set	French external set (n = 32)	TCGA set (n = 29)
Hepatic stem-like	0.84 ± 0.06	0.86 (n = 14)	0.76 (n = 17)
Tumour classical	0.77 ± 0.09	0.88 (n = 3)	n.a. (n = 0)
Inflammatory stroma	0.72 ± 0.10	0.92 (n = 7)	0.80 (n = 5)
Immune classical	0.63 ± 0.08	0.62 (n = 7)	0.78 (n = 5)

CV, cross-validation; n.a., not applicable; TCGA, The Cancer Genome Atlas.

the type of slides (from biopsy or surgical samples, directly or indirectly associated with transcriptomic analysis) on prediction accuracy. Our findings suggest that intratumoural heterogeneity can negatively affect training when non-consecutive slides are used for either molecular profiling or histological analysis. This discrepancy introduces label noise, in that the molecular class we aim to predict may not align with the tissue captured in the image. Although large datasets are generally required for effective neural network training, our results suggest that smaller datasets with high-confidence labels can outperform larger, noise-prone datasets.

Moreover, Kather *et al.*[36] found that flash-frozen slides yielded better performance in molecular prediction tasks within the TCGA dataset, despite their poorer morphological quality compared to FFPE slides. This anomaly could also be attributed to label noise arising from tumour heterogeneity, because the molecular labels in TCGA are extracted from flash-frozen samples. These insights underscore the importance of using consecutive slides for molecular class prediction and could potentially impact the design of future studies. Finally, though more research is needed to validate the clinical use of such predictive models, patient stratification would benefit from combining the prediction of several WSIs sampled in different blocks, which would help capture the main iCCA class of the tumour and help in exploring intratumoural heterogeneity. Indeed, intratumoural molecular heterogeneity in liver cancers, including iCCA, is a key feature that may explain treatment failure and patient prognosis.[37]

Importantly, we included both biopsy and surgical samples in the discovery set to better reflect clinical practice. Even though using biopsies might have reduced our model's performance during cross-validation, it improved performance on the French external validation set. Hence, biopsies may provide complementary information to surgical specimen WSIs (see Fig. 3), and increase the robustness of the trained network. Currently, most AI studies of primary liver cancers have focused on surgical samples,[16,19–21] but most patients with iCCA will not undergo surgery, which may introduce a selection bias. Of note, we found transcriptomic classes differentially represented between surgical and biopsy cases, which highlights the importance of working with both tissue samples. A few studies, mainly focusing on diagnostic tasks, have laid the groundwork for using biopsies and have demonstrated that encouraging deep learning-based results can be obtained in this type of sample despite their size.[18,38,39] Thus, our model could serve as a useful molecular screening tool, particularly for biopsies, where molecular analysis is often limited by the small amount of tissue available.

The main limitation of our study is the unbalanced proportion of each transcriptomic class and, in particular, the rarity of the desert-like class, which represents less than 10% of cases in all sets, precluding the development of a predictive model. Learning from a larger number of cases could be beneficial, but finding complete datasets containing survival and transcriptomic data, and histological slides of FFPE iCCA samples is difficult, as evidenced by the low number of cases available in the TCGA dataset. The methodology we applied for the RNA classifier differed slightly from that described by Martin-Serrano *et al.*,[10] owing to the unavailability of the original code. Nonetheless, the distribution of transcriptomic classes obtained in our analysis was highly consistent with that reported by Martin-Serrano *et al.*,[10] thereby suggesting that both approaches capture the same underlying class structure.

We have developed and validated an SSL model able to predict iCCA transcriptomic classes on WSIs from routine biopsy and surgical samples. This model showed good to very good performance for classifying the four main classes. The ability to predict transcriptomic iCCA classes on routine WSIs could affect patient management by predicting prognosis and guiding the treatment strategy (immunotherapy in inflammatory classes or targeted molecular therapies in hepatic stem-like class).

Affiliations

[1]*AP-HP.Nord, Department of Pathology, FHU MOSAIC, DMU DREAM, Beaujon Hospital, Clichy, France;* [2]*Université Paris Cité, Centre de Recherche sur l'Inflammation (CRI), INSERM, U1149, CNRS, ERL 8252, F-75018 Paris, France;* [3]*Center for Computational Biology (CBIO), Mines Paris, INSERM U900, Institut Curie, PSL University, Paris, France;* [4]*AP-HP, Henri Mondor-Albert Chenevier University Hospital, Department of Pathology, Créteil, France;* [5]*AP-HP.Nord, Department of Molecular Genetics, Bichat Hospital, Paris, France;* [6]*AP-HP.Nord, Department of HPB Surgery & Liver Transplantation, Beaujon Hospital, Université Paris Cité, Clichy, France;* [7]*AP-HP.Nord, Liver Cancer Unit, DMU DIGEST, Beaujon Hospital, 92110, Clichy, France*

Abbreviations

AI, artificial intelligence; FFPE, formalin-fixed paraffin-embedded; HES, hematein eosin saffron; iCCA, intrahepatic cholangiocarcinoma; MIL, multiple instance learning; SSL, self-supervised learning; TGCA, The Cancer Genome Atlas; WSIs, whole slide images.

Financial support

Nuovo-Soldati Foundation, Appel à projet AFEF 2021 grant, SNFGE MAHGE grant.

Conflict of interest

The authors declare no conflicts of interest pertaining to this manuscript.
Please refer to the accompanying ICMJE disclosure forms for further details.

Authors' contribution

Study concept and design (AB, JC, KM, VP), acquisition of data (AB, TL, RN, GL, BP, MA, JA, VP, SL, NTA, JC); analysis and interpretation of data (AB, TL, CP, RN, TW, VP); drafting of the manuscript (AB, TL); critical revision of the manuscript for important intellectual content (ML, MB, KM, TW, VP); statistical analysis (AB, TL); obtained funding (AB); study supervision (TW, VP).

Data availability

The binary classification analysis are available in the online repository: https://github.com/trislaz/ICCA_prediction.
The TCGA-CHOL data including gene expression and WSIs are available from NIH GDC Data Portal (https://portal.gdc.cancer.gov/).
The RNA-seq data have been deposited in Gene Expression Omnibus (GEO) (accession no. GSE244807).

Patient consent statement and ethics approval

Written consent was obtained from all patients as required by French legislation. This study was approved by the local ethics committee (IRB 00006477 no.CER-2022-168).

Acknowledgement

We acknowledge the iGenSeq core facility at ICM for the benefit of its equipment and services, and the Nuovo-Soldati foundation for its support.

Supplementary data

Supplementary data to this article can be found online at https://doi.org/10.1016/j.jhepr.2025.101675.

References

Author names in bold designate shared co-first authorship

[1] Global Burden of Disease Liver Cancer Collaboration, Akinyemiju T, Abera S, Ahmed M, Alam N, Alemayohu MA, et al. The burden of primary liver cancer and underlying etiologies from 1990 to 2015 at the global, regional, and national level: results from the global burden of disease study 2015. JAMA Oncol 2017;3:1683–1691. https://doi.org/10.1001/jamaoncol.2017.3055.

[2] Rahnemai-Azar AA, Weisbrod A, Dillhoff M, et al. Intrahepatic cholangiocarcinoma: molecular markers for diagnosis and prognosis. Surg Oncol 2017;26:125–137. https://doi.org/10.1016/j.suronc.2016.12.009.

[3] Endo I, Gonen M, Yopp AC, et al. Intrahepatic cholangiocarcinoma: rising frequency, improved survival, and determinants of outcome after resection. Ann Surg 2008;248:84–96. https://doi.org/10.1097/SLA.0b013e318176c4d3.

[4] Mavros MN, Economopoulos KP, Alexiou VG, et al. Treatment and prognosis for patients with intrahepatic cholangiocarcinoma: systematic review and meta-analysis. JAMA Surg 2014;149:565–574. https://doi.org/10.1001/jamasurg.2013.5137.

[5] Bridgewater J, Galle PR, Khan SA, et al. Guidelines for the diagnosis and management of intrahepatic cholangiocarcinoma. J Hepatol 2014;60:1268–1289. https://doi.org/10.1016/j.jhep.2014.01.021.

[6] European Association for the Study of the Liver. EASL-ILCA clinical practice guidelines on intrahepatic cholangiocarcinoma. J Hepatol 2023. https://doi.org/10.1016/j.jhep.2023.03.010. S0168-8278(23)00185-X.

[7] Oh D-Y, Ruth He A, Qin S, et al. Durvalumab plus gemcitabine and cisplatin in advanced biliary tract cancer. NEJM Evid 2022;1. https://doi.org/10.1056/EVIDoa2200015. EVIDoa2200015.

[8] **Kelley RK**, **Ueno M**, Yoo C, et al. Pembrolizumab in combination with gemcitabine and cisplatin compared with gemcitabine and cisplatin alone for patients with advanced biliary tract cancer (KEYNOTE-966): a randomised, double-blind, placebo-controlled, phase 3 trial. Lancet Lond Engl 2023;401:1853–1865. https://doi.org/10.1016/S0140-6736(23)00727-4.

[9] Sia D, Hoshida Y, Villanueva A, et al. Integrative molecular analysis of intrahepatic cholangiocarcinoma reveals 2 classes that have different outcomes. Gastroenterology 2013;144:829–840. https://doi.org/10.1053/j.gastro.2013.01.001.

[10] Martin-Serrano MA, Kepecs B, Torres-Martin M, et al. Novel microenvironment-based classification of intrahepatic cholangiocarcinoma with therapeutic implications. Gut 2022. https://doi.org/10.1136/gutjnl-2021-326514. gutjnl-2021-326514.

[11] Moeini A, Sia D, Bardeesy N, et al. Molecular pathogenesis and targeted therapies for intrahepatic cholangiocarcinoma. Clin Cancer Res Off J Am Assoc Cancer Res 2016;22:291–300. https://doi.org/10.1158/1078-0432.CCR-14-3296.

[12] Abou-Alfa GK, Macarulla T, Javle MM, Kelley RK, Lubner SJ, Adeva J, et al. Ivosidenib in IDH1-mutant, chemotherapy-refractory cholangiocarcinoma (ClarIDHy): a multicentre, randomised, double-blind, placebo-controlled, phase 3 study. Lancet Oncol 2020;21:796–807. https://doi.org/10.1016/S1470-2045(20)30157-1.

[13] Abou-Alfa GK, Sahai V, Hollebecque A, et al. Pemigatinib for previously treated, locally advanced or metastatic cholangiocarcinoma: a multicentre, open-label, phase 2 study. Lancet Oncol 2020;21:671–684. https://doi.org/10.1016/S1470-2045(20)30109-1.

[14] Calderaro J, Seraphin TP, Luedde T, et al. Artificial intelligence for the prevention and clinical management of hepatocellular carcinoma. J Hepatol 2022;76:1348–1361. https://doi.org/10.1016/j.jhep.2022.01.014.

[15] Calderaro J, Kather JN. Artificial intelligence-based pathology for gastrointestinal and hepatobiliary cancers. Gut 2021;70:1183–1193. https://doi.org/10.1136/gutjnl-2020-322880.

[16] Kather JN, Calderaro J. Development of AI-based pathology biomarkers in gastrointestinal and liver cancer. Nat Rev Gastroenterol Hepatol 2020;17:591–592. https://doi.org/10.1038/s41575-020-0343-3.

[17] Chen X, Wang X, Zhang K, et al. Recent advances and clinical applications of deep learning in medical image analysis. Med Image Anal 2022;79:102444. https://doi.org/10.1016/j.media.2022.102444.

[18] Albrecht T, Rossberg A, Albrecht JD, et al. Deep learning-enabled diagnosis of liver adenocarcinoma. Gastroenterology 2023. https://doi.org/10.1053/j.gastro.2023.07.026. S0016-5085(23)04883-04887.

[19] Saillard C, Schmauch B, Laifa O, et al. Predicting survival after hepatocellular carcinoma resection using deep-learning on histological slides. Hepatol Baltim Md 2020. https://doi.org/10.1002/hep.31207.

[20] **Zeng Q**, **Klein C**, Caruso S, et al. Artificial intelligence predicts immune and inflammatory gene signatures directly from hepatocellular carcinoma histology. J Hepatol 2022;77:116–127. https://doi.org/10.1016/j.jhep.2022.01.018.

[21] Cheng N, Ren Y, Zhou J, et al. Deep learning-based classification of hepatocellular nodular lesions on whole-slide histopathologic images. Gastroenterology 2022;162:1948–1961.e7. https://doi.org/10.1053/j.gastro.2022.02.025.

[22] Shi J-Y, Wang X, Ding G-Y, et al. Exploring prognostic indicators in the pathological images of hepatocellular carcinoma based on deep learning. Gut 2021;70:951–961. https://doi.org/10.1136/gutjnl-2020-320930.

[23] Yamashita R, Long J, Saleem A, et al. Deep learning predicts postsurgical recurrence of hepatocellular carcinoma from digital histopathologic images. Sci Rep 2021;11:2047. https://doi.org/10.1038/s41598-021-81506-y.

[24] Campanella G, Hanna MG, Geneslaw L, Miraflor A, Werneck Krauss Silva V, Busam KJ, et al. Clinical-grade computational pathology using weakly supervised deep learning on whole slide images. Nat Med 2019;25:1301–1309. https://doi.org/10.1038/s41591-019-0508-1.

[25] Lazard T, Lerousseau M, Decencière E, et al. Giga-SSL: self-supervised learning for gigapixel images. 2022. https://doi.org/10.48550/arXiv.2212.03273.

[26] Bedossa P, Poynard T. An algorithm for the grading of activity in chronic hepatitis C. The METAVIR Cooperative Study Group. Hepatol Baltim Md 1996;24:289–293. https://doi.org/10.1002/hep.510240201.

[27] Dobin A, Davis CA, Schlesinger F, et al. STAR: ultrafast universal RNA-seq aligner. Bioinforma Oxf Engl 2013;29:15–21. https://doi.org/10.1093/bioinformatics/bts635.

[28] Liao Y, Smyth GK, Shi W. featureCounts: an efficient general purpose program for assigning sequence reads to genomic features. Bioinforma Oxf Engl 2014;30:923–930. https://doi.org/10.1093/bioinformatics/btt656.

[29] Ilse M, Tomczak JM, Welling M. Attention-based deep multiple instance learning [n.d].

[30] Lazard T, Bataillon G, Naylor P, et al. Deep learning identifies morphological patterns of homologous recombination deficiency in luminal breast cancers from whole slide images. Cell Rep Med 2022;3:100872. https://doi.org/10.1016/j.xcrm.2022.100872.

[31] He K, Zhang X, Ren S, Sun J. Deep residual learning for image recognition. 2015. https://doi.org/10.48550/arXiv.1512.03385.

[32] Lubuela G, Beaufrère A, Albuquerque M, et al. Prognostic impact of the tumour microenvironment in intrahepatic cholangiocarcinoma: identification of a peritumoural fibro-immune interface. Virchows Arch Int J Pathol 2024. https://doi.org/10.1007/s00428-024-03922-5.

[33] Li B, Eliceiri KW. Dual-stream maximum self-attention multi-instance learning, 2020. https://doi.org/10.48550/arXiv.2006.05538; 2020.

[34] Tellez D, Litjens G, Bándi P, et al. Quantifying the effects of data augmentation and stain color normalization in convolutional neural networks for computational pathology. Med Image Anal 2019;58:101544. https://doi.org/10.1016/j.media.2019.101544.

[35] Marini N, Otalora S, Wodzinski M, et al. Data-driven color augmentation for H&E stained images in computational pathology. J Pathol Inform 2023;14:100183. https://doi.org/10.1016/j.jpi.2022.100183.

[36] Kather JN, Heij LR, Grabsch HI, et al. Pan-cancer image-based detection of clinically actionable genetic alterations. Nat Cancer 2020;1:789–799. https://doi.org/10.1038/s43018-020-0087-6.

[37] **Ma L**, **Wang L**, **Khatib SA**, et al. Single-cell atlas of tumor cell evolution in response to therapy in hepatocellular carcinoma and intrahepatic cholangiocarcinoma. J Hepatol 2021;75:1397–1408. https://doi.org/10.1016/j.jhep.2021.06.028.
[38] Pantanowitz L, Quiroga-Garza GM, Bien L, et al. An artificial intelligence algorithm for prostate cancer diagnosis in whole slide images of core needle biopsies: a blinded clinical validation and deployment study. Lancet Digit Health 2020;2:e407–e416. https://doi.org/10.1016/S2589-7500(20)30159-X.
[39] **Xu F**, **Zhu C**, **Tang W**, et al. Predicting axillary lymph node metastasis in early breast cancer using deep learning on primary tumor biopsy slides. Front Oncol 2021;11:759007. https://doi.org/10.3389/fonc.2021.759007.

Keywords: intrahepatic cholangiocarcinoma; Transcriptomic classes; self-supervised learning; histological slide.

Received 29 June 2025; received in revised form 20 October 2025; accepted 5 November 2025; Available online 11 November 2025

Journal of Hepatology, Volume *8*

Supplemental information

Self-supervised learning to predict intrahepatic cholangiocarcinoma transcriptomic classes on routine histology

Aurélie Beaufrère, Tristan Lazard, Rémy Nicolle, Gwladys Lubuela, Jérémy Augustin, Miguel Albuquerque, Baptiste Pichon, Camille Pignolet, Victoria Priori, Nathalie Théou-Anton, Mickael Lesurtel, Mohamed Bouattour, Kévin Mondet, Jérôme Cros, Julien Calderaro, Thomas Walter, and Valérie Paradis

Self-supervised learning to predict intrahepatic cholangiocarcinoma transcriptomic classes on routine histology

Aurélie Beaufrère, Tristan Lazard, Rémy Nicolle, Gwladys Lubuela, Jérémy Augustin, Miguel Albuquerque, Baptiste Pichon, Camille Pignolet, Victoria Priori, Nathalie Théou-Anton, Mickael Lesurtel, Mohamed Bouattour, Kévin Mondet, Jérôme Cros, Julien Calderaro, Thomas Walter, Valérie Paradis

Table of contents

Material and methods 2

Table S1 3

Table S2 4

Table S3 5

Table S4 6

Table S5. 7

Fig. S1 8

Fig. S2 9

Fig. S3. 10

Fig. S4 11

Material and methods

Slide preprocessing and tessellation

Slides from the discovery set were stained with HES and encoded in svs format. Slides from the external French validation set were stained with HES and encoded in ndpi format. Slides from the TCGA validation set were stained with hematoxylin-eosin (HE) and encoded in svs format. Tissue regions automatically extracted using Otsu thresholding were then exhaustively split into 2899811 patches of 224×224 pixels (without overlapping) at 10x using the OpenSlide library in Python.

We present the results in the discovery set according to the following three pre-processing protocols with or without extraction of the region of interest (ROI), each requiring varying levels of expert pathologist involvement (Figure 1, Fig. S3):

1. No-filter (∅): all tiles including tumour and non-tumour are processed as they are (encompassing both tumour and non-tumour regions).

2. Manual-filter (M): an expert pathologist (AB) extensively annotates tumour regions using ImageScope software, from which patches are extracted.

3. Learning-filter (A): tiles are filtered using logistic regression trained on a dataset of 3000 tile embeddings, randomly extracted and labelled by an expert pathologist (AB).

Table S1. List of morphological criteria assessed by the expert pathologist for all cases of the three datasets

Histological criteria	Assessment
**Tumour grade**	Classification into well, moderately or poorly differentiated tumour according to the 5th edition of the WHO classification
**Tumour histological type**	Small duct, large duct or other subtypes
**Necrosis**	Percentage
**Tumour fibrosis**	Semi quantitatively assessed, classified into three classes: no or mild, moderate and intense
**Immune tumour infiltration**	Semi quantitatively assessed, classified into four classes: no inflammation, low, moderate and high
**Tertiary lymphoid structure (TLS)**	Presence or absence

Table S2. Repartition of the five transcriptomic classes in the discovery set and in the two external validation sets.

Transcriptomic groups	Total n=307 (%)	Discovery set n=246 (%)	French external validation set n = 32 (%)	TCGA set n = 29 (%)
**Hepatic stem-like**	121 (39)	90 (37)	14 (44)	17 (59)
**Desert like**	14 (5)	11 (4)	1 (3)	2 (7)
**Tumor classical**	37 (12)	34 (14)	3 (9)	0 (0)
**Immune classical**	69 (22)	57 (23)	7 (22)	5 (17)
**Inflammatory stroma**	66 (21)	54 (22)	7 (22)	5 (17)

Table S3. Repartition of the five transcriptomic classes according to the type of samples in the discovery set.

Transcriptomic groups	Total n=246 (%)	Surgical samples n=109 (%)	Biopsies n = 137 (%)	*p*
**Hepatic stem-like**	90 (37)	53 (49)	37 (27)	**<0.001**
**Desert like**	11 (4)	4 (4)	7 (5)	0.759
**Tumor classical**	34 (14)	16 (15)	18 (13)	0.853
**Immune classical**	57 (23)	15 (14)	42 (31)	**0.002**
**Inflammatory stroma**	54 (22)	21 (19)	33 (24)	0.439

Table S4. Model performance for the hepatic stem-like binary classification task on external validation sets

Validation dataset	AUC score	Balanced accuracy score	F1 score
**TCGA set**	0.76	0.73	0.74
**French external set**	0.86	0.73	0.71

Area under the curve, AUC; The cancer genome atlas, TCGA

Table S5. Effects of the ROI extraction method on the external validation performances for the Hepatic stem-like binary classification task.

The same method is applied on both the training and validation datasets. On the TCGA set, no distinct advantage is observed for method A over M or vice versa, as it varies based on the metric under consideration. In the French external set, manually segmenting the tumour seems to be advantageous. Nevertheless, in both datasets, using an ROI extraction method is more effective than not using any.

**Validation dataset**	**ROI extraction method**	**AUC score**	**Balanced accuracy score**	**F1 score**
**TCGA set**	ø	0.74	0.60	0.61
	M	0.76	**0.73**	**0.74**
	**A**	**0.82**	0.64	0.63
**French external set**	ø	0.78	0.71	0.68
	**M**	**0.86**	**0.73**	**0.71**
	A	0.8	0.73	0.72

Area under the curve, AUC; Learning-Filter, L; Manual-Filter, M; No-Filter, ∅; Region of interest, ROI

Fig. S1. Histological features of iCCA

A. Example of small duct type iCCA

B. Example of large duct type iCCA

C. Example of an iCCA with an abundant fibrous stroma

D. Example of an iCCA with an abundant inflammatory stroma containing tertiary lymphoid structures

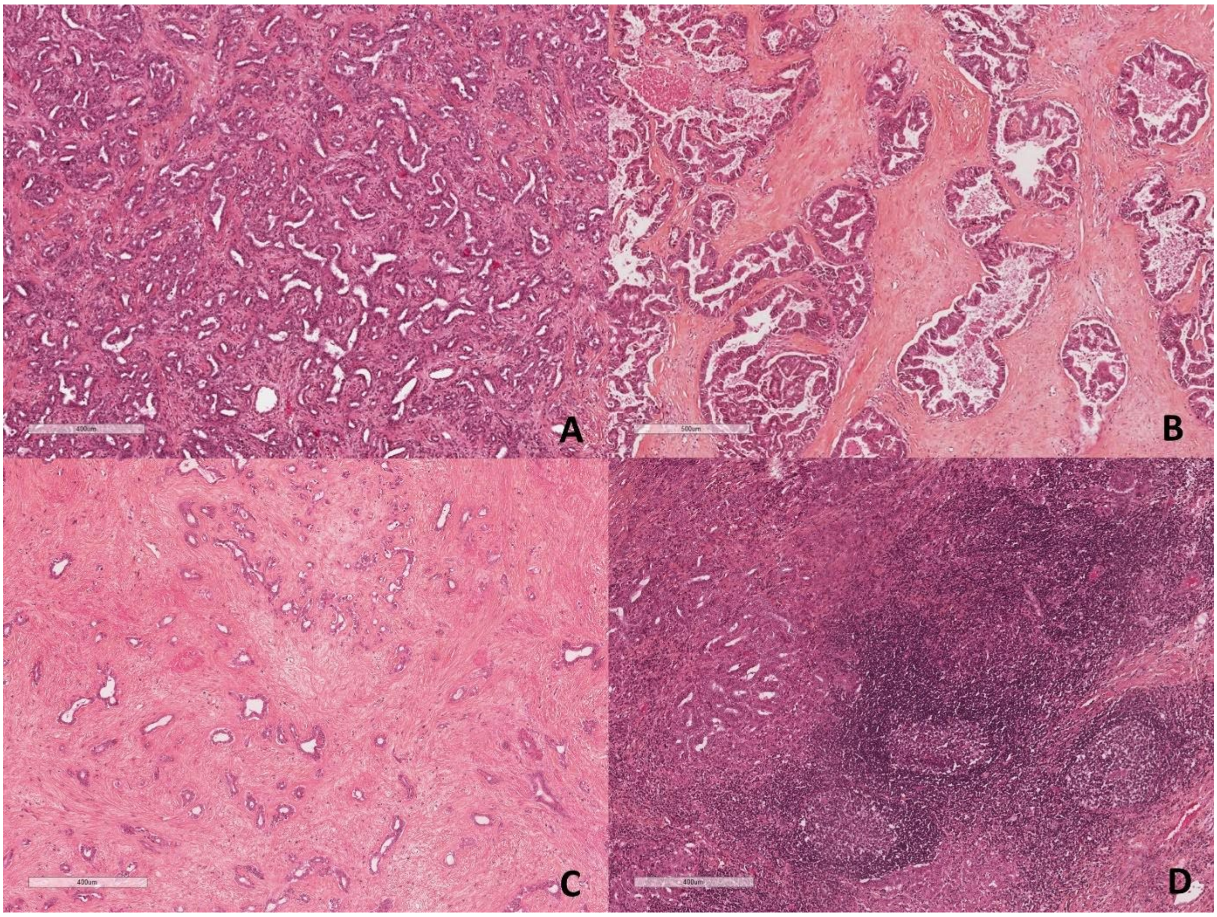

Fig. S2. Type of slides including in the study.

For surgical specimens, slides corresponding to blocks used for transcriptomic analysis in both the discovery set and the French external set were labeled as surgical slides (S RNA+). In the discovery and the TCGA sets, slides from blocks not used for transcriptomic analysis were labeled (S RNA-). For biopsy cases, the FFPE block used for RNA sequencing corresponded directly to the selected slide, which was labeled (B RNA+).

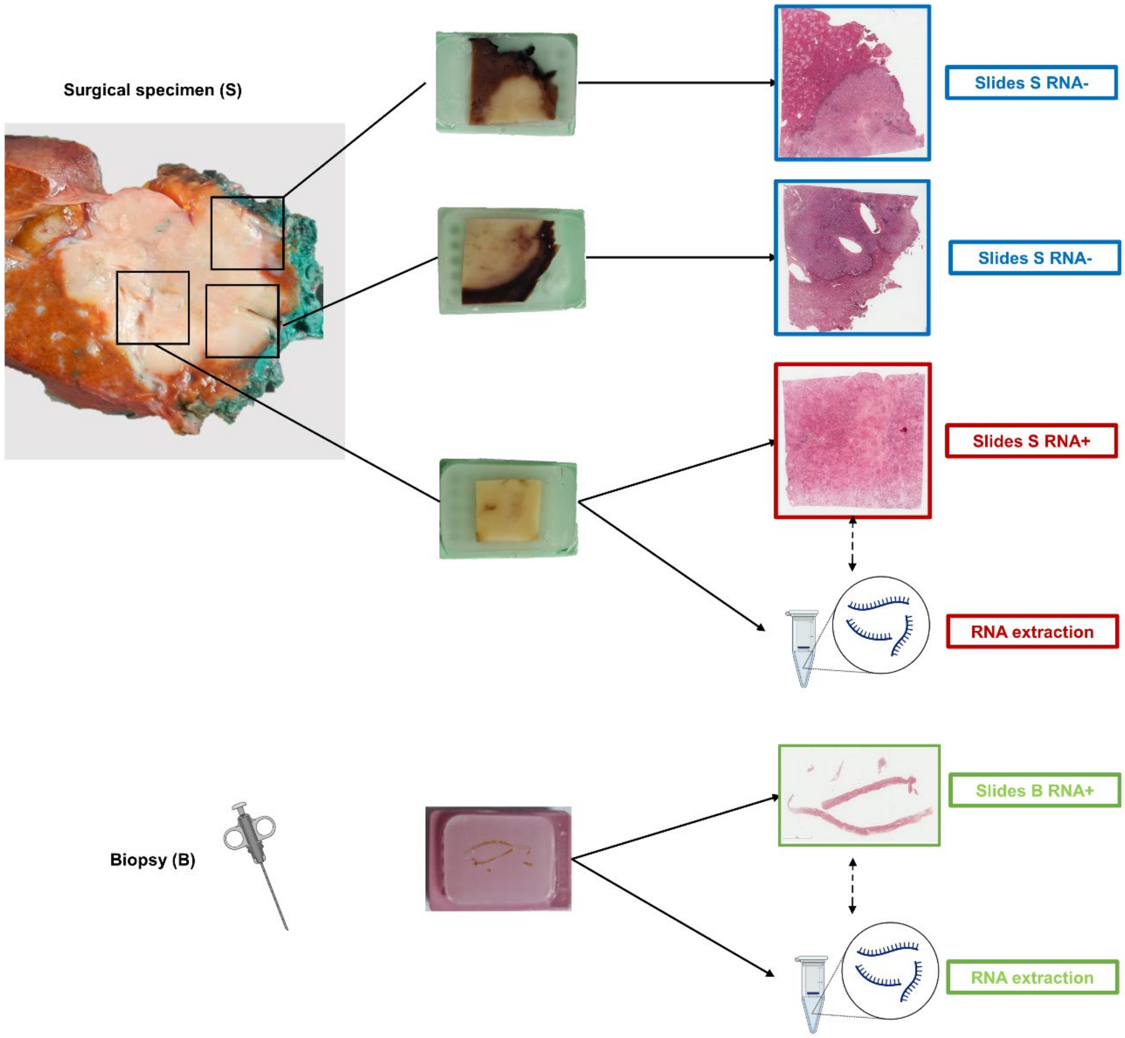

Fig. S3. Three different pre-processing protocols with or without extraction of region of interest (ROI).

- No-Filter (Ø): All tiles including tumour and non-tumour are processed as they are, encompassing both tumour and non-tumour regions.
- Manual-Filter (M): An expert pathologist (AB) extensively annotates tumour regions using ImageScope software, from which patches are extracted.
- Learning-Filter (A): Tiles are filtered using a logistic regression trained on a dataset of 3000 tile embeddings, randomly extracted and labelled by an expert pathologist (AB).

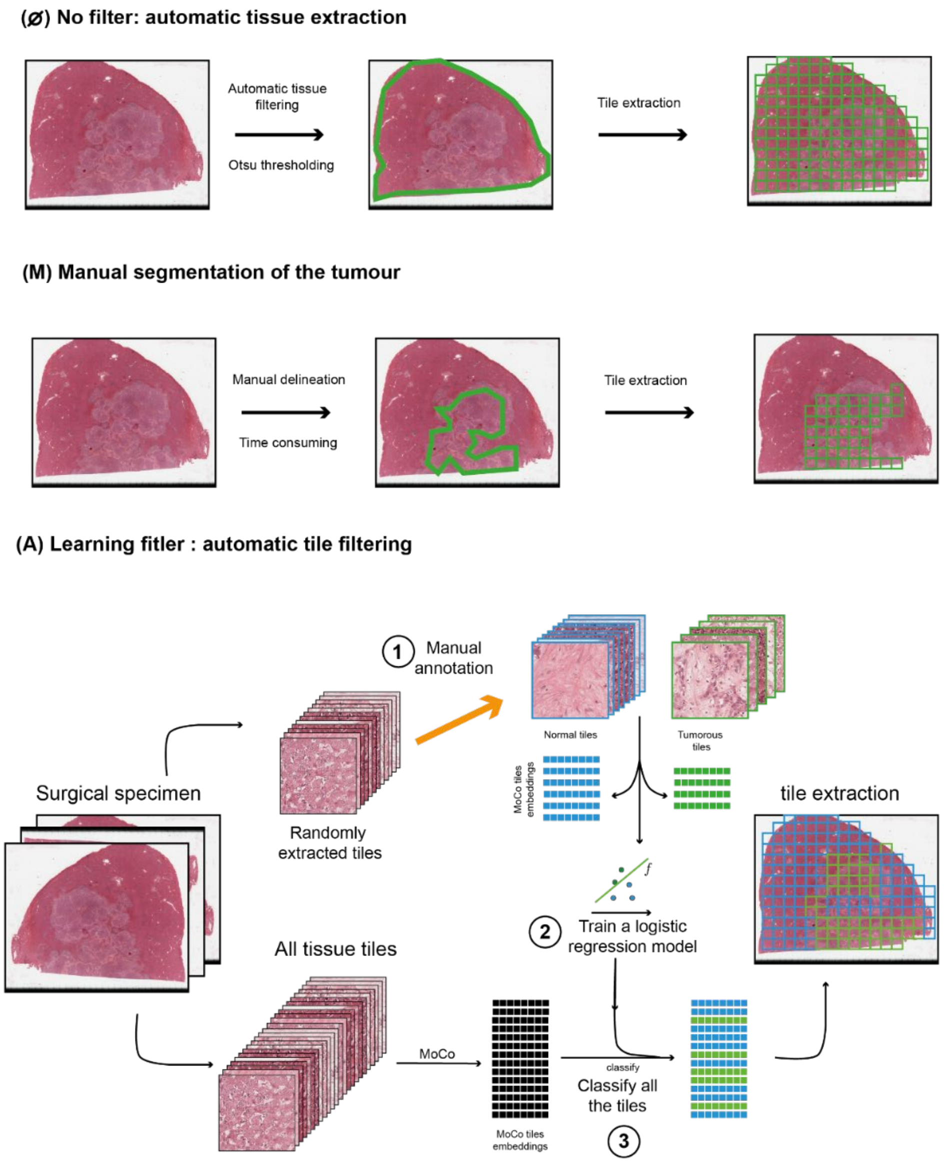

Fig. S4. Histological iCCA subtypes (large vs small duct) according to the five transcriptomic groups

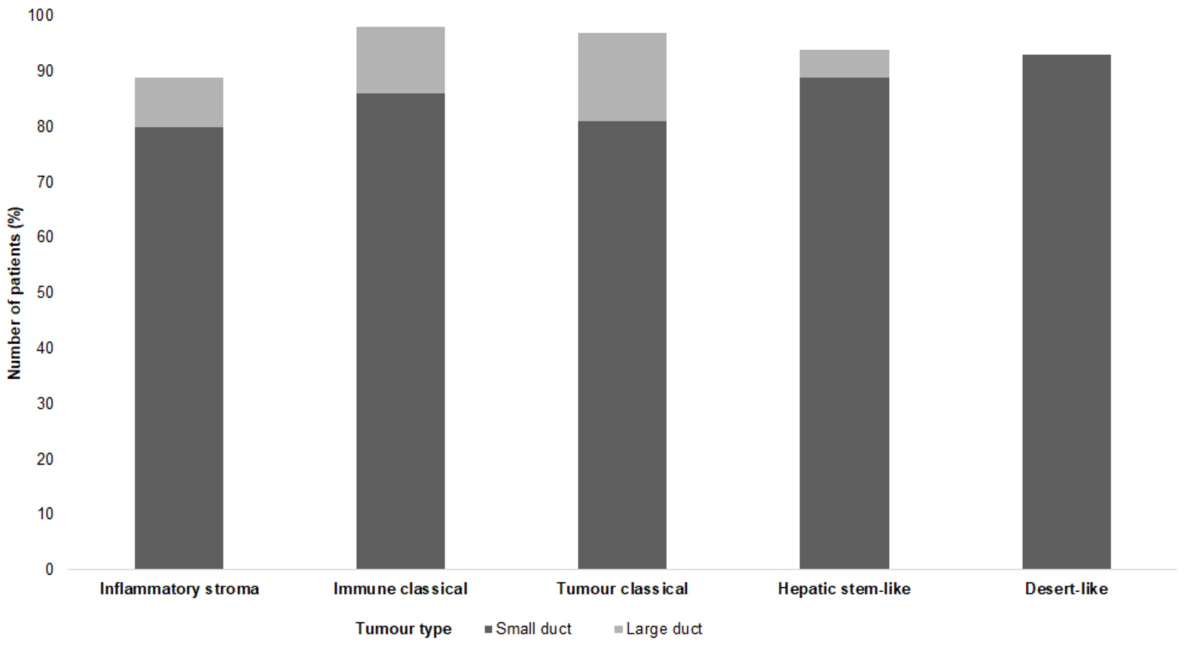

Supplement: Multimedia component 4 [file mmc4.pdf]
